# Variation in adherence measures as a function of calculation methods

**DOI:** 10.3389/fphar.2024.1460327

**Published:** 2024-11-13

**Authors:** Jeffrey M. Rohay, Jacqueline M. Dunbar-Jacob

**Affiliations:** University of Pittsburgh, School of Nursing, Pittsburgh, PA, United States

**Keywords:** adherence, electronic monitoring, operational definition, calculation method, within patient variability

## Abstract

**Aim:**

We aim to compare different operational definitions of medication adherence as well as examine the within-patient variability among these measures among patients treated for multiple comorbid conditions.

**Methods:**

Electronically monitored adherence data from a study on comorbid conditions were examined using three different calculation methods. DAILY adherence calculated the number of administrations divided by the number prescribed, without considering inter-dose interval. TIMING used predefined inter-dose intervals. Measures were aggregated to six 30-day periods. A PILLCOUNT approach counted the total administrations divided by the expected number in each 30-day period. Within-patient variability was computed based on DAILY and TIMING results for each 30-day period.

**Results:**

Results varied by adherence calculation method. PILLCOUNT demonstrated the largest adherence rates (89%–92%); DAILY rates were lower (79%–85%); and TIMING was the lowest (62%–68%) over the 6-month period. TIMING within-patient variability (29%–35%) was larger than DAILY (20%–25%).

**Discussion:**

Differences among the three methods confirm the importance of the adherence definition. TIMING may underestimate medicinal effects because patients may take medication as instructed (e.g., with meals) rather than at fixed intervals. PILLCOUNT may overestimate adherence by not accounting for inconsistent use. DAILY may best provide daily estimates of correct administration. Higher variability for TIMING may indicate patients are more likely to vary time between doses. Adherence calculation methods are important in interpreting results. Variability measures provide a more complete picture of adherence and may raise the likelihood of effects on biological outcomes. We propose studies of adherence include calculation method in the definition of adherence.

## 1 Introduction

Adherence to prescribed medication has been intensively studied since the late 1960s. Reviews consistently find wide ranges of adherence rates across populations. In 1966, Milton Davis, a medical sociologist, reported that a literature review indicated 15%–93% of patients failed to adhere to medical prescriptions (Davis, 1966). Since that time studies continue to show wide ranges of adherence to medications. In 2024, Gaujoux-Viala et al. reported adherence among persons with rheumatoid arthritis ranged from 30% to 80% (Gaujoux-Viala et al., 2024). Dugunchi et al. (2024) reported non-adherence in coronary artery disease ranged from 33% to 55% (Dugunchi et al., 2024). Using claims and EHR data, Finlayson et al. (2024) reported variations in adherence across medications for persons with diabetes ranging from 52.4% for combination medications to 73.7% for amylin analogs, suggesting one source of variability may be the type of medication (Finlayson et al., 2024). Time may be another factor. In a longitudinal study of adherence following a myocardial infarction, just 29% of patients were adherent for the full year of the study while the average adherence for drug ranged from 62% to 67%, suggesting significant variability within individual patients (Pietrzykowski et al., 2020).

One issue in finding a broad range in adherence is the method of measurement that is used in studies. Davis himself utilized physician questionnaires to determine patient adherence. A study by Roth & Caron (1978) found that physician estimates tended to be inaccurate and that patients were highly variable over time in their medication taking as well as inaccurate in their estimates when compared with medication bottle counts (Roth and Caron, 1978). Since that time numerous studies have reported inconsistencies between adherence reports from differing measures. For example, Alili et al. reported the median adherence overestimate of self-reported adherence was 17% compared with electronically monitored adherence (Alili et al., 2016). In general, these studies have shown higher self-reported adherence rates when compared with objective measures, including medication monitors, pharmacy refills, pill counts, and visual analog scales (Atkinson et al., 2016). Monnette et al. reported a range of −66.3 to 61.5 difference between two self-report and monitoring devices (Monnette et al., 2018). Indeed, a review of self-report measures among cardiovascular populations found none of the existing PROMs (patient reported outcome measures) were recommended for use based upon measurement properties (Oliveira et al., 2023). Further, the ability of measures to detect clinical changes varies, as evidenced by a study by Dunbar-Jacob, et al. which found that electronic monitors and only the Shea, of multiple self-report assessments, predicted cholesterol lowering from use of statins (Dunbar-Jacob et al., 2013). Subsequent investigation by Dunbar-Jacob & Rohay indicated that self-report and electronic assessment (Medication Event Monitoring System [MEMS]) identified different and independent predictors of adherence among individuals assessed at the same time for the same drug (Dunbar-Jacob and Rohay, 2016). Overall adequate psychometric testing of adherence measures is poor, but where conducted are found to be low in sensitivity and specificity (Konstantinou et al., 2022).

Underlying various methods of measuring adherence are the variety of concepts and definitions used in defining adherence as well as the variety of cut points utilized in defining non-adherence or acceptable adherence. Thus, measures may address such issues as the number of pharmacy refills within a specific time period, the patient estimate of how frequently they take their medication as prescribed, the electronic record of accessing a medication, the count of medications missing from a bottle over a specified duration of time, the patient report of their beliefs and/or confidence in taking their medication, the time from onset of medication taking to stopping, each of which may be operationally defined in a different manner. Aremu et al. note that medication adherence can be defined as “the act or extent of conforming to a provider recommendation/prescription based on timing, dosage, and frequency of medication use … (and) as a ratio of the number of drug doses taken to the number of doses prescribed over a given period” (Aremu et al., 2022). Few studies provide such a concise definition, if any definition is provided at all. Shah, Touchette, & Marrs (2023) provide a detailed review of these variations. Each of these methods is likely to yield differing estimates of adherence (Shah et al., 2023).

Many of these methods and definitions do not adhere to the commonly accepted definition of adherence suggested by Haynes in 1979 (Haynes, 1979) and modified by the WHO in 2003 to include “the extent to which a person’s behavior, taking medication, following a diet, and/or executing lifestyle changes, corresponds with agreed recommendations from a healthcare provider” (Sabate, 2003). Further complicating the problems contributing to variability in estimates of levels of adherence is the variability in defining the threshold for satisfactory adherence. A systematic review by Baumgartner et al. revealed the highly varied methods of calculating adherence precluded the possibility of identifying a threshold level for good (clinically effective) adherence (Baumgartner et al., 2018).

These variations in adherence assessments lead to significant challenges in the development of systematic reviews to identify effective interventions as well as predictors or factors associated with adherence. It is the aim of this study to demonstrate the effect of different operational definitions of medication adherence on adherence findings and to examine the within person variability, relatively unexamined, in adherence across measures among patients with co-morbid type 2 diabetes, hypertension, and hyperlipidemia.

## 2 Methods

Data from the Diabetes Comorbidity study (NIDDK R01 DK59048) designed to improve medication adherence among patients with diabetes were examined to determine the effect that adherence calculation method has on the interpretation of results. Participants in the study were being treated for three chronic conditions–diabetes, hypertension, and hypercholesterolemia. This report focuses on adherence to then diabetes and hypertension medications these participants were prescribed. Medication adherence was monitored for one medication for each of the three conditions using the MEMS (Haberer and Gellman, 2004; Aardex). The MEMS system incorporates a microchip in the pill bottle cap that recorded the date and time that the cap was removed and replaced on the bottle. This recording served as a presumptive medication taking event. Participants were randomized to one of three treatment conditions – (Intervention, Intervention plus maintenance, and Usual Care). Participants completed assessments at baseline, 6 months, and 12 months. The MEMS system was used continuously during the baseline period and for the 12-month follow-up period. To evaluate just the effect of calculation method and not effects due to the intervention, only those participants randomized to usual care were considered in the analyses. Data were divided into six 30-day intervals and MEMS data were used to create six monthly sets of computations. Additionally, only participants on once-per-day, twice-per-day, or three-per-day regimens were included.

Electronically monitored adherence was calculated using three different methods. The first method examined was similar to a pill count (PILLCOUNT) in which the number of administrations observed in the 30 days interval was divided by the number expected regardless of when they occurred. Next, we examined the average daily adherence (DAILY). DAILY was calculated based on the number of administrations observed on a given day divided by the number prescribed. However, the inter-dose interval was not considered. DAILY was then summed and divided by the total days of observation for an overall adherence rate. The final method (TIMING) used predefined inter-dose intervals in determining the DAILY measure to account for both missed doses and consecutive doses with relatively short inter-dosing intervals (e.g., <2 h).

In all cases, we imposed a behavioral penalty for “over adherence” by “folding” the adherence measure–for example, someone on a twice per day regimen who had 3 events recorded would have an initial adherence rating of 150%. However, they would be penalized for the amount over 100% - i.e., 50% - for a final adherence measure of 50% (100%–50%). For PILLCOUNT this was done on the monthly measure. For DAILY and TIMING adherence this was done on the DAILY measure. The DAILY and TIMING adherence measures were then aggregated into six 30-day intervals by calculating the average adherence rate over the 30 days. Additionally, we assessed within-patient variability by calculating each participant’s monthly standard deviation for their DAILY and TIMING adherence measures.

Self-reported adherence, based on the Morisky 4-item Scale (MMAS) (Krapek et al., 2004; Zillich et al., 2005) and the response to the single question *How much of the time do you follow the instructions about when and how much of your medication you should take* at 6 months was also examined.

Analyses were completed using MEMS adherence ratings for both diabetes and hypertension medications. To assess the impact of computational method on health outcome, electronically monitored diabetes adherence rates were examined by level of HBA1c control - good control defined as HBA1c less than or equal to 7; at risk defined as HBA1c between 7 and 9; and poor control defined as HBA1c greater than or equal to 9 (NCQAa). Similarly, electronically monitored hypertension adherence rates were examined by level of blood pressure control - good control defined as systolic blood pressure less than or equal to 120 and diastolic blood pressure less than or equal to 80; at risk systolic blood pressure between 120 and 140 or diastolic blood pressure between 80 and 90; and poor control defined as systolic blood pressure greater than 140 or diastolic blood pressure greater than 90 (NCQAb). Descriptive statistics, including means, standard deviations, and confidence intervals for adherence measures, and frequency counts for categorial variables were used. Computations and analyses were conducted using SAS software v9.4 (SAS Software, 2023).

## 3 Results

Sixty-eight participants randomized to the usual care group from the Diabetes Comorbidity Study provided MEMS adherence data for both their diabetes and hypertension medications. Participants were diagnosed with diabetes on average 10.7 years prior to the study and were diagnosed with hypertension on average 13.4 years prior to the study. These participants were also assessed for disease control using HBA1c for diabetes and blood pressure for hypertension at baseline and 6 months. They were predominately female (59%) and had mean age of 63.9 (SD = 10.6). Of the 68 participants 59 provided HBA1c data at 6 months. Of these, 11 (19%) were in good diabetic control. Fifty participants provided blood pressure data at 6 months. Of these, 12 (24%) were in good hypertensive control.

Average electronic adherence measures varied by adherence calculation method (see Table 1). Diabetes adherence calculated using the PILLCOUNT method demonstrated the largest adherence rates, averaging 91.2% (SD = 9.9%) and ranging from 94% to 98% over the 6-month periods. DAILY adherence rates were lower, averaging 83.3% (SD = 17.0%) and ranging from 76% to 80%. TIMING was the lowest, averaging 64.7% (SD = 21.6%) and ranging from 59% to 64% over the 6-month period.

**TABLE 1:** Adherence rates by computational method and disease outcome.

DIABETES									
	PILLCOUNT			DAILY			TIMING		
Month	Good control	At risk	Poor control	Good control	At risk	Poor control	Good control	At risk	Poor control
1	91.6%	93.9%	92.2%	83.6%	86.1%	88.6%	66.3%	68.7%	60.3%
2	88.1%	92.1%	91.7%	80.5%	88.5%	82.8%	66.4%	63.3%	58.8%
3	89.3%	90.0%	88.4%	82.9%	74.8%	80.8%	67.3%	60.1%	53.2%
4	94.1%	92.9%	91.3%	83.6%	83.8%	86.5%	65.1%	67.2%	57.9%
5	93.0%	90.1%	91.7%	87.0%	79.1%	87.9%	73.4%	64.2%	64.3%
6	92.0%	92.3%	89.8%	85.8%	79.1%	86.1%	68.1%	63.7%	57.5%
HYPERTENSION									
1	96.5%	96.0%	96.4%	91.1%	86.1%	93.2%	80.9%	75.4%	83.0%
2	96.3%	97.5%	91.3%	93.6%	88.5%	81.5%	66.8%	70.1%	62.8%
3	97.4%	97.4%	95.0%	89.8%	74.8%	80.0%	74.9%	67.9%	67.7%
4	97.2%	99.3%	96.2%	90.4%	83.8%	87.0%	74.2%	68.3%	77.9%
5	96.6%	95.9%	96.3%	91.3%	79.1%	79.5%	75.4%	73.0%	73.0%
6	97.9%	98.3%	97.3%	94.4%	79.1%	81.5%	77.1%	72.9%	74.8%

**FIGURE 1:**
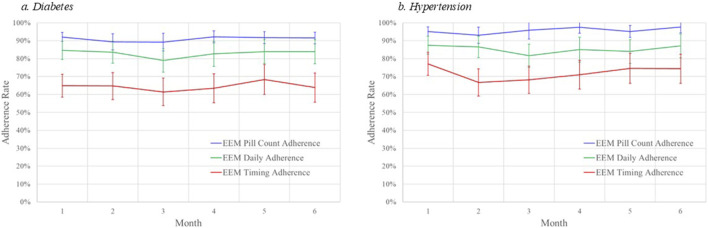
Monthly adherence rates by computational method and disease. **(A)** diabetes. **(B)** hypertension.

The diabetes adherence rates for each computational method were relatively stable over the 6 months (see Figure 1A). PILLCOUNT adherence rates were approximately 90% in each of the 6 months while DAILY adherence rates averaged about 7 percentage point lower at approximately 83%. TIMING was significantly lower than both, at about 65% over the 6 months.

For comparison, self-report adherence based on the MMAS (4-item) was 85% at 6 months. Furthermore, 74% of the participants reported that they usually followed the instructions for their prescribed medication.

Within-participant variability for diabetes medication adherence was larger for TIMING, averaging 32% (range 29%–35%) while DAILY averaged 21% (range 20%–25%). TIMING variability declined slightly by about 0.9% per month over the 6 months from 34.7% to 31.5%. DAILY variability was more stable, declining about 0.6% per month from 23.4% to 20.5%.

Results were comparable when examining adherence to hypertension medications. Average hypertension medication electronic adherence measures varied by adherence calculation method. Adherence calculated using the PILLCOUNT method demonstrated the largest adherence rates, averaging 95.1% (SD = 6.8%) and ranging from 93% to 98% over the 6-month periods. DAILY adherence rates were lower, averaging 84.8% (SD = 15.8%) and ranging from 82% to 88%. TIMING was the lowest, averaging 71.7% (SD = 21.4%) and ranging from 67% to 77% over the 6-month period.

The hypertension medication adherence rates for each computational method were relatively stable over the 6 months (see Figure 1B). PILLCOUNT adherence rates were approximately 95% in each of the 6 months while DAILY adherence rates averaged about 10 percentage points lower at approximately 85%. TIMING was significantly lower than both, at about 72% over the 6 months.

Within-participant variability for hypertension medication adherence was larger for TIMING, averaging 33% (range 29%–39%) while DAILY averaged 24% (range 21%–27%). TIMING variability declined slightly by about 1.1% per month over the 6 months from 38.8% (Month 2) to 28.7%. DAILY variability was more stable, declining about 0.1% per month from 22.9% to 21.0%.

**FIGURE 2:**
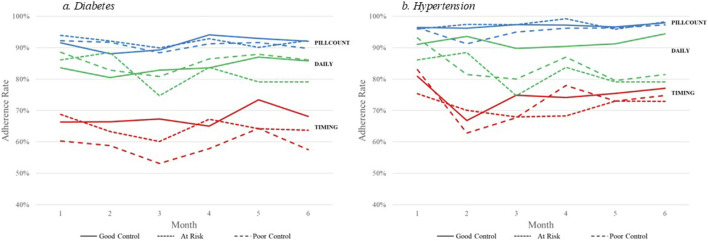
Electronic medication adherence by disease outcome computational method. **(A)** diabetes. **(B)** hypertension.

We also examined level of disease control by adherence computational method. Figure 2A displays the adherence rates by PILLCOUNT, DAILY, and TIMING for participants in good control, at risk, and in poor control of their diabetes based on level of HBA1c at 6 months. Figure 2B displays the adherence rates by PILLCOUNT, DAILY, and TIMING for participants in good control, at risk, and in poor control of their hypertension based on levels of systolic and diastolic blood pressure at 6 months.

For diabetes, PILLCOUNT did not discriminate between disease control among the participants, with adherence rates around 90% across the three HBA1c outcome groups. The DAILY adherence computational method displayed some discrimination among the HBA1c groups. Lower adherence rates were observed for the DAILY computational method for those at risk in Month 5 and Month 6. More pronounced differences were observed for the TIMING computation method over all 6 months, with participants in poor control of HBA1c demonstrating consistently lower adherence rates.

For hypertension, again PILLCOUNT did not discriminate between disease control among the participants, with adherence rates around 95% across the three blood pressure outcome groups. The DAILY adherence computational method displayed some discrimination among the blood pressure groups. Lower adherence rates were observed for the DAILY computational method for those at risk and in poor control over the 6-month period. Only minor differences were observed for the TIMING computation method over the 6 months.

For diabetes, adherence levels were comparable within calculation method, with PILLCOUNT demonstrating the largest adherence rates, followed by DAILY, and then TIMING. To identify differences in the predictive ability of the methods, we compared the poor control group (HBA1c ≥ 9) to those not in poor control (HBA1c < 9). We conducted a Receiver Operating Characteristic (ROC) analysis with adherence levels at 3 months used to evaluate the sensitivity and specificity (See Table 2) of calculation method to predict diabetes control. The TIMING method performed best for predicting poor control (Area Under the Curve [AUC] = 0.63) with a sensitivity of 0.80 and specificity of 0.61. DAILY and PILLCOUNT did not perform as well with AUCs around 50% (0.53 and 0.54 respectively).

**TABLE 2:** Area under the curve, sensitivity and specificity by calculation method.

	Diabetes				Hypertension			
Method	N	AUC	Sensitivity	Specificity	N	AUC	Sensitivity	Specificity
Timing	46	0.63	0.80	0.61	41	0.56	0.40	0.71
Daily	46	0.53	0.50	0.72	41	0.65	0.60	0.65
Pill Count	46	0.54	0.60	0.61	41	0.55	0.36	0.88
Variability								
Timing	50	0.61	0.64	0.62	46	0.55	0.60	0.50
Daily	50	0.49	0.45	0.64	46	0.70	0.80	0.56

For hypertension, again adherence levels were comparable within calculation method, with PILLCOUNT demonstrating the largest adherence rates, followed by DAILY, and then TIMING. Again, ROC analyses were conducted to determine if differences in the predictive ability of the methods were present for hypertension. The poor control group (Systolic BP ≥ 140 or Diastolic BP ≥ 90) was compared to those not in poor control (Systolic BP < 140 and Diastolic BP < 90). For comparability we used adherence levels at 3 months to predict hypertension control. Here the TIMING method performed best for predicting poor control (AUC = 0.65) with a sensitivity of 0.60 and specificity of 0.65. TIMING and PILLCOUNT did not perform as well with AUCs around 50% (0.56 and 0.55 respectively).

Variability measures differed between the DAILY adherence method and the TIMING adherence method for both Diabetes adherence and Hypertension adherence, with the TIMING method demonstrating larger variability. ROC analyses using variability were similar to those using adherence measurements. For Diabetes, TIMING variability performed better (AUC = 0.61; Sensitivity = 0.64; Specificity = 0.62) than DAILY variability (AUC = 0.49; Sensitivity = 0.45; Specificity = 0.64). For hypertension, DAILY variability performed better (AUC = 0.70; Sensitivity = 0.80; Specificity = 0.56) than TIMING variability (AUC = 0.55; Sensitivity = 0.60; Specificity = 0.50; see Table 2).

## 4 Discussion

This study is unique in the process of examining assessment of adherence. The same subjects with the same diagnoses were assessed over the same time period using the same measurement strategy, the MEMs electronic monitor of adherence, to determine the degree of adherence to their medication for co-occurring type 2 diabetes and hypertension. For each subject, adherence was calculated in three ways, the overall percent of prescribed medication taken (PILLCOUNT), the percent of days in which the medication was taken as prescribed (DAILY), and the percent of doses taken within a window that maximized coverage by the medications (TIMING). This simple act of altering the method of calculation resulted in differences in the reported levels of adherence. The differences were substantial. For example, the average adherence calculated for the diabetes medication was 90% (PILLCOUNT), 83% (DAILY), and 65% (TIMING). The average adherence calculated for the hypertension medication was 95% (PILLCOUNT), 85% (DAILY), and 72% (TIMING). The self-report measures, which asked participants to consider both medications, yielded 6 month estimates of 85% for the MMAS-4 and 74% for the question regarding the amount of time the participant took the medications as prescribed. Thus, it is probable that much of the wide variation seen in reported medication adherence in the literature may be due to the method of calculating adherence, the potential lack of precision in self-reported measures due a small number of possible adherence rates, as well as the measurement strategy, utilized in studies.

The importance of adherence is in its role in leading to good clinical outcomes. Thus, it is important in medication efficacy studies (are the results due to the degree of adherence or to the level of efficacy of the medication), in studies designed to improve clinical outcomes, as well as in clinical practice to obtain the best outcomes for each patient. In each of these cases, the detection of poor adherence is important. Once again, this study showed that within a measurement strategy the method of calculating adherence yielded varying levels of sensitivity and specificity in the detection of good or poor control. For the diabetes medication, sensitivity was 0.80 for TIMING, 0.50 for DAILY, and 0.60 for PILLCOUNT and for hypertensive medication was 0.40 for TIMING, 0.60 for DAILY, and 0.36 for PILLCOUNT. Thus, the ability of adherence values to identify those with poor control also varied by calculation methods. The ability of adherence values to identify those with good clinical control also varied. Specificity in detecting good control in diabetes was 0.61 for TIMING, 0.72 for DAILY, and 0.61 for PILLCOUNT while in hypertension it was 0.71 for TIMING, 0.65 for DAILY, and 0.88 for PILLCOUNT. Thus, the relationship between measured adherence and disease control varied by method of calculation of adherence.

These data suggest that calculation methods within measures are important in the interpretation of studies and individual cases using adherence data to account for level of medication taking. Variability measures also provide a more complete picture of adherence and may raise the likelihood of effects on biological outcomes. Within participant variability was able to be determined within the DAILY and TIMING methods of calculation. For the diabetes medication the average variability was 32% for TIMING and 21% for DAILY. For the hypertension medication the average variability was 33% for TIMING and 24% for DAILY. Thus, there was considerable variability detected in the taking schedule for medication using both TIMING and daily estimates. Once again, sensitivity and specificity of the variability measures varied by calculation method. For diabetes, sensitivity for TIMING was 0.64 and for daily was 0.45 while for hypertension it was 0.60 for TIMING and 0.80 for daily. Specificity for diabetes was 0.62 for TIMING and 0.64 for daily while for hypertension it was 0.50 for TIMING and 0.56 for daily.

Results generated by the different computational methods impact the interpretation of results. While adherence rates were relatively stable, regardless of computational methods, the method used did impact how the level of adherence would be gauged with more complex computational methods resulting in lower adherence rates. The relationship between computational method and disease differed between diabetes and hypertension, even though this was the same person during the same time period taking both drugs. When examining disease outcomes, in this case HBA1c control, little difference was noted among those with good control (HBA1c < 7, at risk (HBA1c between 7 and 9), and those with poor control (HBA1c > 9) based on PILLCOUNT and DAILY calculations. However, when TIMING was incorporated, those in poor control demonstrated lower adherence rates. However, it should be noted that the participants in this study took different diabetes medications, but the sample size precluded medication specific analyses.

Not only do the different operational definitions reflect differences in adherence but also differences in the identified predictors of adherence. Previous work by Dunbar-Jacob & Rohay identified different and independent predictors of higher adherence based on self-report (MMAS) and electronic assessment (MEMS) measures among individuals assessed at the same time for the same drug (Dunbar-Jacob and Rohay, 2016). The chosen method of calculation may vary dependent upon the interest of the investigator or clinician as well as characteristics of the medication and the disease. For example, is the investigator interested in a medication with a long half-life with little effect of variability on efficacy or on a medication with a shorter half-life leading to an effect of variability in taking patterns on efficacy? This study suggests that different information may be gleaned from the different calculation methods. Further there is some additional support for the variation in concordance between measurement methods dependent upon the calculation method. In this study, self-report measures in hypertension appeared to be most closely aligned with 6-month average daily (MMAS-4), 85% and 85% respectively, and TIMING (frequency of adherence question), 72% and 74% respectively, while in diabetes the alignment appeared to be strongest between daily and the MMAS-4 (83% and 85%) and the TIMING and single question (65% and 74%). On the other hand, the MMAS-4 of 85% does not compare well with 6-month TIMING adherence (diabetes – 65%; hypertension – 72%) nor does the single question of 72% compare well with the 6-month PILLCOUNT (90% & 95%) or the 6-month DAILY (83% & 85%).

Thus, the estimation of participant or patient adherence is complex. There is significant variability in estimates between measures and between calculation methods within measures. It is important to understanding the meaning of adherence in any study as well as the conduct of systematic reviews that there is a complete description of the operational definition of adherence, including not only the measurement method but the method of calculation as well. Because the calculation method reveals different elements of the participant/patient’s medication taking behavior, an understanding of the calculation and its meaning is important to the assessment of the clinical case and the design of remedial efforts as well. To properly characterize differences (i.e., a difference in the operational definition of adherence v. Difference in interventions), we strongly recommend that studies of adherence provide a description of the calculation method utilized to estimate adherence along with the method of measurement used for assessment.

## Data Availability

The datasets presented in this article are not readily available because Data are available by request if they meet the requirements of the informed consent signed by each participant. Requests to access the datasets should be directed to dunbar@pitt.edu.
